# Case report: coexistence of primary hyperparathyroidism with giant toxic nodular goiter

**DOI:** 10.1186/s12902-022-01117-0

**Published:** 2022-08-09

**Authors:** Wei Zhang, Fangyi Liu, Kang Chen, Yajing Wang, Jingtao Dou, Yiming Mu, Zhaohui Lyu, Li Zang

**Affiliations:** 1 Department of Endocrinology, The First Medical Center of PLA General Hospital, 100853 Beijing, China; 2 Department of Endocrinology, Affiliated People’s Hospital, Zhejiang Provincial People’s Hospital, Hangzhou Medical College, Zhejiang Province 310003 Hangzhou, China; 3 Department of Interventional ultrasound, The First Medical Center of PLA General Hospital, 100853 Beijing, China

**Keywords:** Toxic nodular goiter, PHPT, PTH, SPECT/CT, Thermal ablation

## Abstract

**Background:**

The coexistence of primary hyperparathyroidism (PHPT) and giant toxic nodular goiter is very rare. Moreover, PHPT could be easily overlooked because hyperthyroidism may also lead to hypercalcemia. A 99mTc-MIBI scan of the parathyroid glands is often negative when they are concomitant.

**Case presentation:**

Here, we report a rare case of the coexistence of giant toxic nodular goiter and PHPT that had been ignored for many years but was successfully treated with an ultrasound-guided parathyroid adenoma microwave ablation (MWA).

**Conclusion:**

Reoperation for PHPT carries an increased risk of cure failure and complications. Thermal ablation has been proven effective in inactivating hyperfunctioning parathyroid lesions and in normalizing both serum parathyroid hormone (PTH) and calcium.

## Background

The concomitant rate of primary hyperparathyroidism (PHPT) with hyperthyroidism is much higher than previously reported. Hyperthyroidism can lead to abnormal bone metabolism. Therefore, when these patients present with hypercalcaemia and osteoporosis, it is easy to ignore the possibility of PHPT. Moreover, PHPT combined with toxic nodular goitre is very rare, and MIBI scans fail to accurately locate the primary site in such cases.

Here, we report a hyperthyroid patient presenting with cervical enlargement hypercalcaemia and severe osteoporosis for more than 10 years. The biochemical assays revealed hypercalcaemia (2.76 mmol/L), hypophosphatemia (0.70 mmol/L), and elevated parathyroid hormone (PTH) levels (134.60 pg/mL). The patient was then diagnosed with toxic nodular goitre with PHPT. The coexistence of PHPT had been ignored for many years, which led to bone fractures and recurrent urinary stones. Due to the giant nodular goitre, the MIBI scan could not locate the diseased parathyroid gland before the first operation. To successfully treat the diseased parathyroid sites, SPECT-CT combined with thyroid ultrasound and contrast-enhanced ultrasound (CEUS) was performed for precise positioning.

## Case presentation

In November 2021, a 58-year-old Chinese man was admitted to our hospital for hyperthyroidism with thyroid nodules for more than 10 years and a high plasma calcium concentration for 2 years.

In 2010, the patient went to the local hospital for a weight loss of approximately 15 kg within six months and neck enlargement. He was diagnosed with hyperthyroidism based on elevated levels of thyroid hormone and decreased levels of thyroid stimulating hormone (TSH). Thyroid ultrasound suggested thyroid nodules, and thyrotropin receptor antibody (TRAb) was not detected. Then, he was treated with methimazole 10 mg once a day. However, his use of the medication and follow-ups with a doctor were irregular, and his neck gradually enlarged. In addition, he did not undergo a plasma calcium test at that time.

**Fig. 1:**
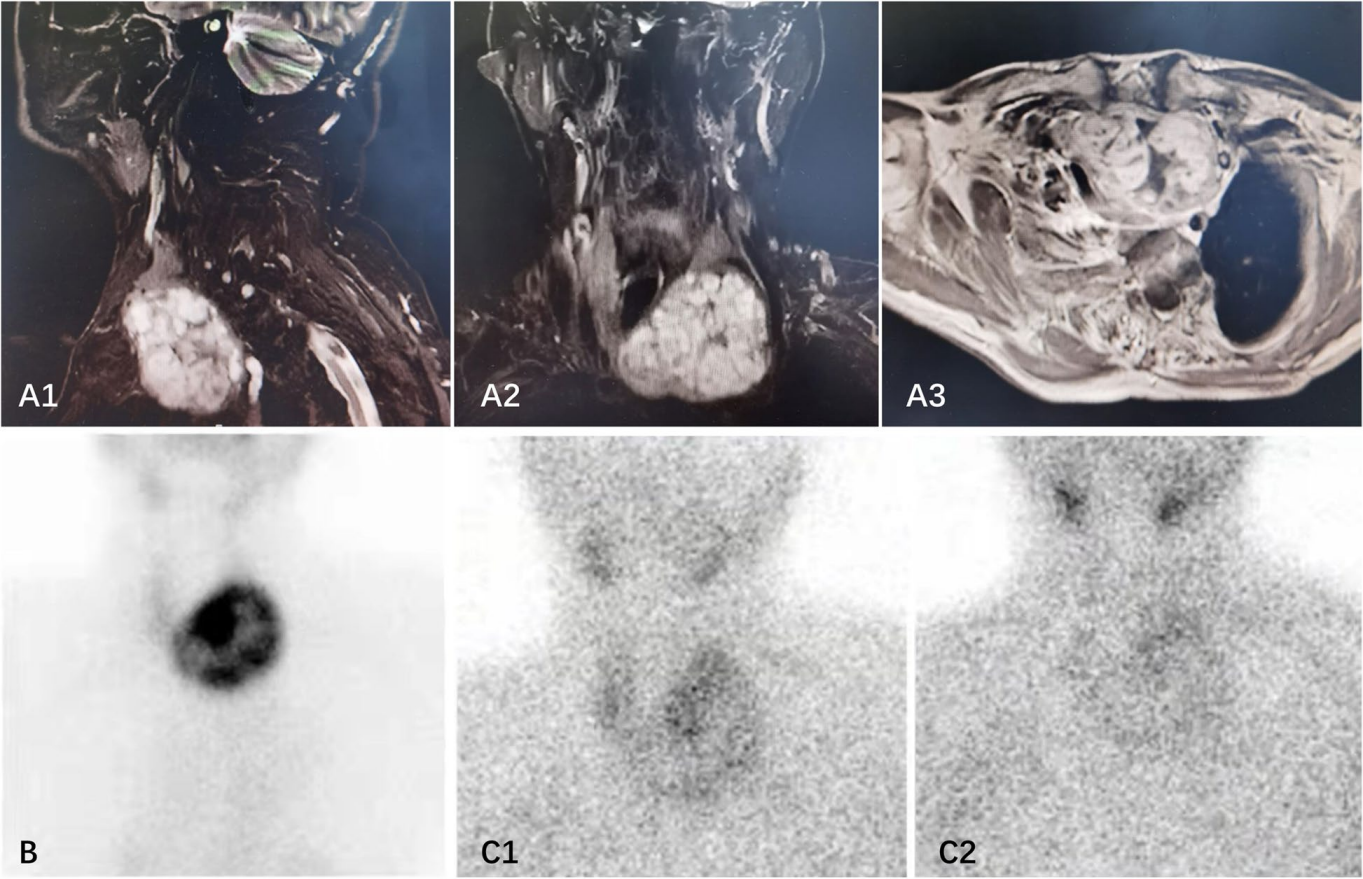
** A** Neck MRI showing a hyperdense lesion in the left lower thyroid gland, and trachea displacement to right side; **B** ^99m^TcO4 scintigraphy: A focus of increased uptake at the left thyroid pole, and multiple ‘hot’ thyroid nodules in the left and isthmus of thyroid gland, making the consideration of giant thyroid goiter; **C** 30 min after intravenous injection with 99MTC-MIBI showed abnormal appearance and left thyroid gland enlargement. 2 h later, there was a slightly hyperactive area in the left lower thyroid gland

In 2019, he visited our outpatient department for an aggravated uncomfortable feeling around the cervical region and hoarseness. Biochemical assays revealed hyperthyroidism with an FT4 level of 49.79 pmol/L (normal range 10.42–24.32), an FT3 level of 7.82 pmol/L (normal range 2.76–6.3), and a TSH level of 0.01 mU/L (normal range 0.35–5.5), and he was negative for TRAb. Laboratory examination also implied primary hyperparathyroidism (PHPT) with an elevated plasma calcium level of 3.24 mmol/L (normal range 2.09–2.54), an elevated parathyroid hormone (PTH) level of 90.78 pg/mL (normal range 15–65), and a decreased phosphorus level of 0.62 mmol/L (normal range 0.89–1.6). Thyroid ultrasound and magnetic resonance imaging (MRI) showed a giant nodular goitre (81*56*69 mm) in the left thyroid lobe with intrathoracic invasion, and the trachea was displaced to the right side. However, ultrasound did not suggest any parathyroid lesions. Radionuclide 99 m-TcO4 thyroid imaging suggested high-functioning lesions based on multithyroid nodules in the left thyroid lobe. 99mTc sesta-MIBI scintigraphy further showed homogeneous tracer uptake similar to the findings from radionuclide 99 m-TcO4 thyroid imaging. Furthermore, no other abnormal or ectopic uptake were observed (Fig. 1), which suggests the coexistence of a toxic nodular goitre with PHPT. Because no other parathyroid lesions were found, he was advised to undergo left hemithyroidectomy. However, the operation was postponed by his subsequent myocardial infarction. Therefore, he received methimazole treatment, and his thyroid hormone level was maintained within the normal range. In June 2021, he underwent a subtotal left thyroidectomy in the department of otolaryngology of our hospital, and the histopathology showed benign nodular goitre.

Additionally, he underwent parathyroid gland exploration, and no exact lesion was found. Euthyroidism was achieved, and methimazole treatment was stopped after the operation. Nevertheless, the laboratory tests showed a constantly high level of PTH and plasma calcium and a low level of phosphorus three days after surgery, which indicated the persistent existence of hyperparathyroidism.

**Fig. 2:**
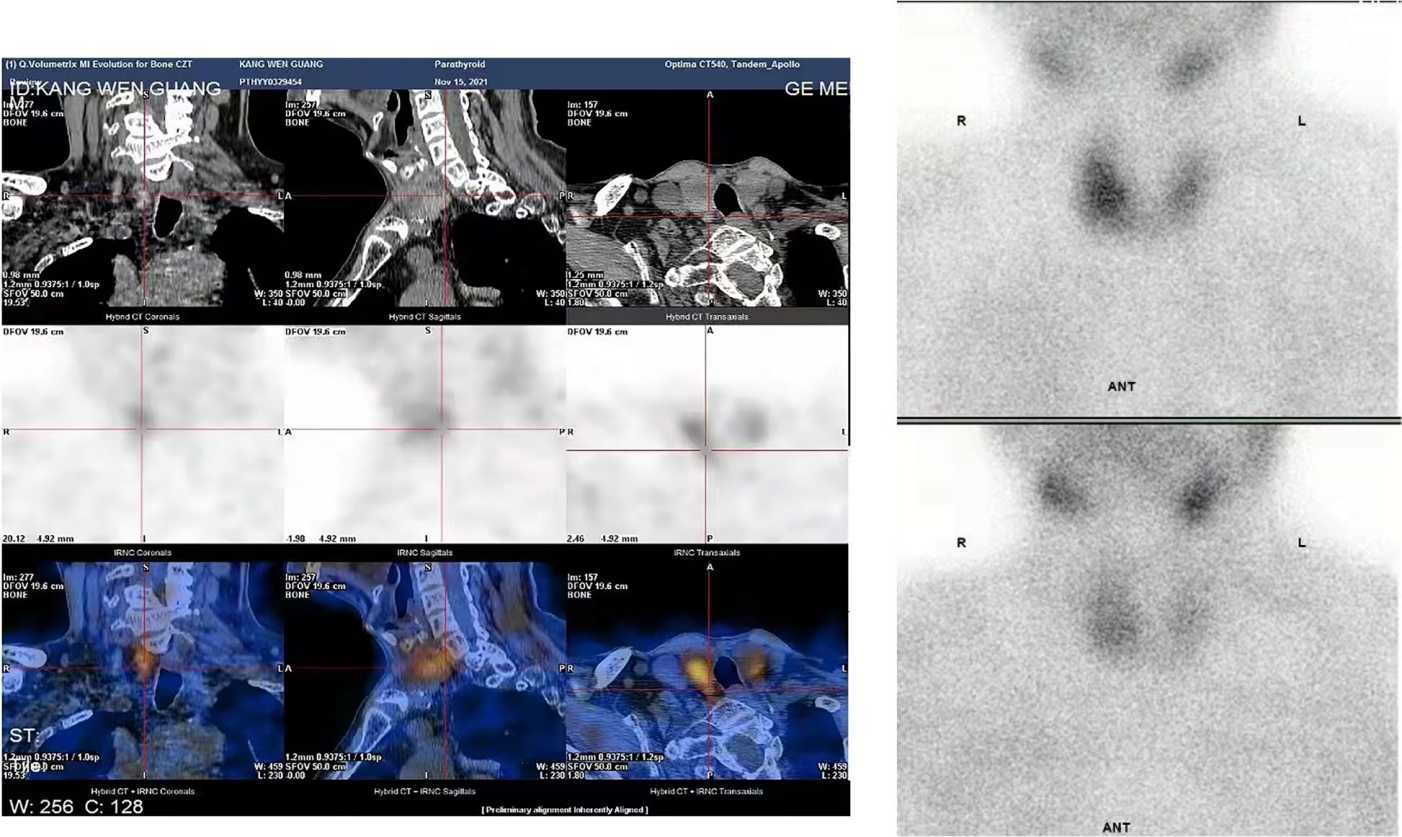
30 min after intravenous injection with 99mTc-MIBI showing an enlarged right lobe of thyroid and a slight hyperactive zone in the middle and lower part. 2 h later, there was persistent radioactive focal in the middle and lower part of right lobe. A SPECT scan revealed an enlarged thyroid gland in the right lobe, with a focal area (7.2 × 8 mm) in the middle and lower part

In October 2021, he returned to our department because of complaints of fatigue, tachycardia, loose teeth, scoliosis, and joint pain caused by the unrelieved hyperparathyroidism. Additionally, he had suffered from recurrent renal colic due to urinary stones for many years. He also had a history of severe osteoporosis and hip arthroplasty in 2013 because of a left femoral neck low trauma fracture. We reviewed his medical records from 2013, and the laboratory examination revealed hypercalcaemia as well as hypophosphatemia; however, PTH was not tested at that time. After admission, the biochemical assays revealed hypercalcaemia (2.76 mmol/L), hypophosphatemia (0.70 mmol/L), elevated PTH levels (134.60 pg/mL), reduced 25-hydroxyvitamin D3 levels (15.2 ng/mL; normal range 20–32) and normal levels of 24-hour urinary calcium (3.64 mmol/24 h). The bone mineral density test suggested severe osteoporosis (T-score: hip − 4.0, vertebra − 3.3), and bone X-ray showed subperiosteal bone resorption with decreased bone density and arthritic changes. However, his thyroid ultrasound did not show any remarkable signs of parathyroid lesions. Neck computed tomography (CT) revealed a suspected parathyroid nodule (15*10 mm) behind the left lobe of the thyroid. In contrast, single-photon emission computed tomography combined with computed tomography (SPECT/CT) showed increased tracer uptake in the lower medium of the right thyroid lobe (7.2*8 mm) (Fig. 2). Therefore, he was diagnosed with PHPT without a definite location of the pathogenic lesion, considering the controversial imaging results by CT and SPECT/CT.

**Fig. 3:**
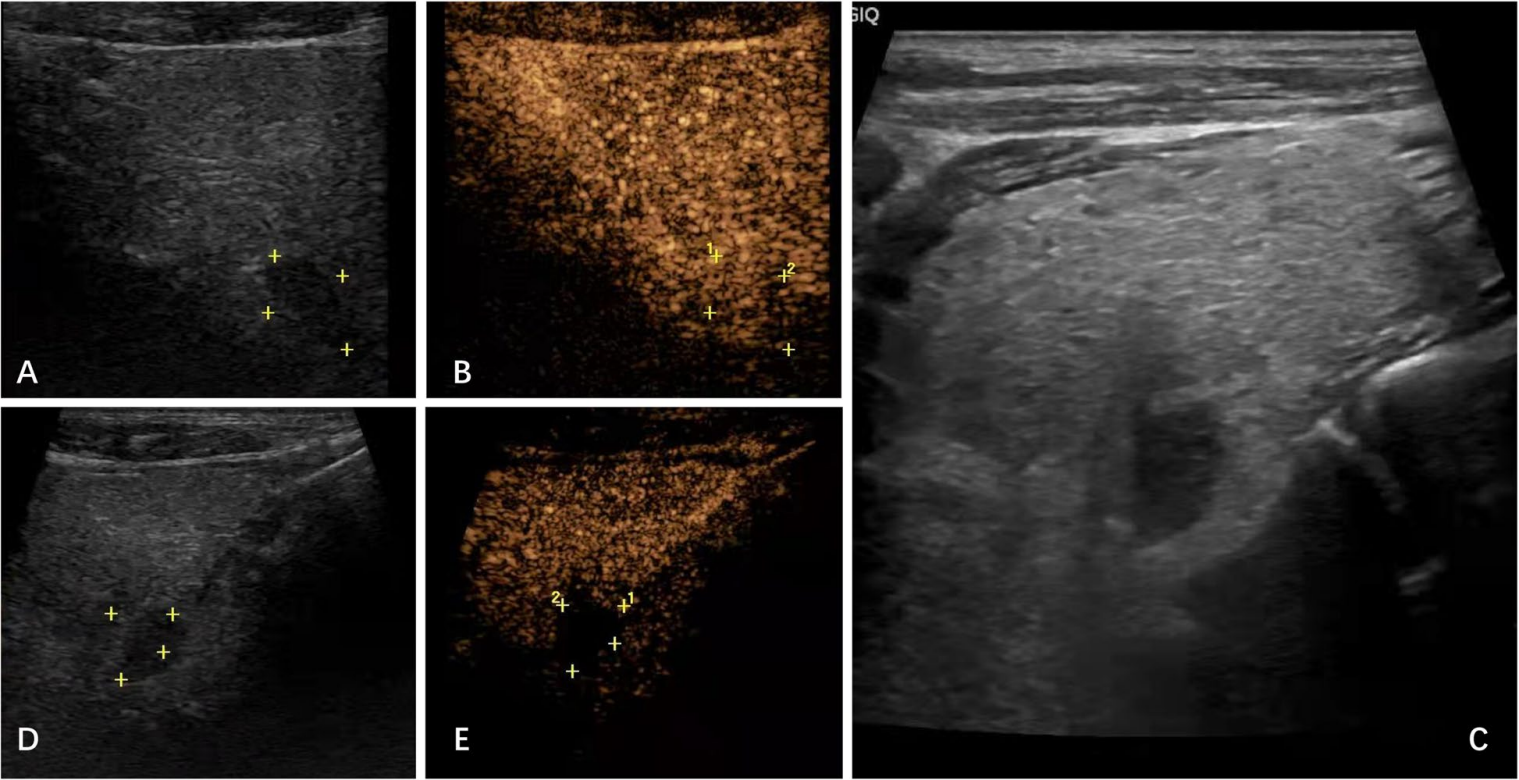
**A** Gray scale ultrasonography of neck before ablation (Color box placed in the suspected parathyroidoma lesion); **B** Neck CEUS before ablation showed continuous enhanced lesion (1.4cmx1.0cmx1.0 cm); **C** Ultrasonography visualization during ablation; **D** Neck Gray scale ultrasonography visualization after ablation; **E** Neck CEUS after ablation

**Fig. 4:**
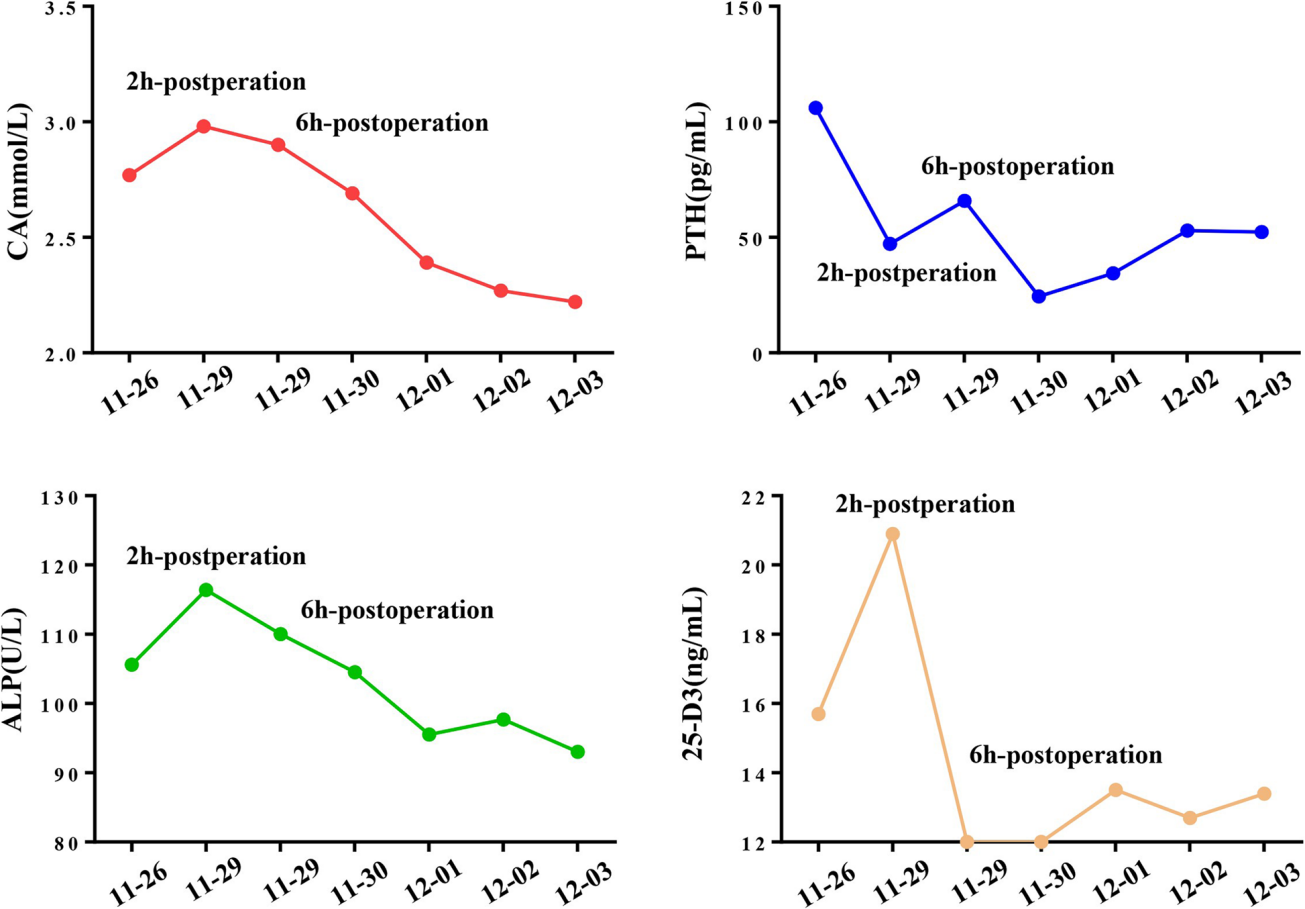
Gradual variation of serum calcium, PTH, ALP and 25-D3 during the post-operation

We performed an MRI scan. This scan indicated that the left nodule was postoperative residual thyroid tissue, and no other parathyroid lesions were found. Thyroid ultrasound was performed again, and a suspected nodule of the parathyroid gland in the right thyroid lobe was ultimately found based on the SPECT/CT results (Fig. 3A). Contrast-enhanced ultrasound (CEUS) revealed a nodule rich in blood supply behind the middle right lobe of the thyroid, which was considered a parathyroid adenoma (Fig. 3B). Finally, the patient was treated with ultrasound-guided parathyroid adenoma microwave ablation (MWA) (Fig. 3C), and the nodule was necrotic 3 days later (Fig. 3D, E). His PTH recovered 2 h after treatment and remained within the normal range for the next 5 days. However, his PTH levels and plasma vitamin D and calcium levels fluctuated slightly. His plasma calcium and ALP levels returned to normal 3 days after the operation and continued to decrease. Furthermore, no hypocalcaemia or related symptoms occurred (Fig. 4). Finally, the patient was administered calcitriol 0.25 µg and calcium carbonate 600 mg twice a day after discharge to prevent hypocalcaemia and osteoporosis. He was then followed-up in our outpatient department. The laboratory results showed that PTH was 48.2 pg/ml, plasma calcium was 2.24 mmol/L and 25-hydroxyvitamin D3 was 15.2 ng/ml after one month, and PTH was 42.7 pg/ml, plasma calcium was 2.19 mmol/L and 25-hydroxyvitamin D3 was 17.9 ng/ml after three months (Table 1). These findings indicate a reasonable response to our therapeutic approach. No familial hyperparathyroidism syndrome was detected in this patient.

**Table 1:** Timeline of patient biochemical characteristics

Subject	FT3 (2.76-6.3pmol/L)	FT4 (10.42–24.32 pmol/L)	TSH (0.35-5.5mU/L)	CA (2.09-2.54mmol/L)	P (0.89-1.6mmol/L)	PTH (15-65pg/mL)	25-(OH) D3 (20–32 ng/mL)	
**2010**	Elevated thyroid hormone and decreased TSH level			NA				
**2013**	NA			NA				
**2019**	7.82	49.79	0.01	3.24	0.62	90.78	NA	
**2021.06** (Pre-operation)	5.04	13.05	1.63	2.75	0.76	140.60	16.90	
**2021.07** (Post-operation)	3.80	13.26	3.22	2.83	0.66	125.00	14.70	
**2021.10**	5.58	21.52	0.49	2.76	0.70	134.60	15.20	
**2021.11.** (Pre- MWA)	NA			2.77	0.77	106	15.7	
**2021.11** (2 h Post-MWA)	NA			2.98	0.79	47.23	20.9	
**2022.01** (1month after MWA)	NA			2.24	0.97	48.2	15.2	
**2022.03** (3 month after MWA)	5.13	22.8	1.03	2.19	1.08	42.7	17.9	

*Abbreviation: MWA *Microwave ablation

## Discussion and conclusions

Approximately 17–84% of patients suffering from PHPT have concomitant thyroid disease [1, 2]. Wagner et al. reported a high occurrence of primary hyperparathyroidism in patients with thyroid disease (0.29%) compared with those without thyroid dysfunction (0.09%) [3]. Castellano et al. reported that among 238 PHPT patients undergoing parathyroidectomy, 118 patients had coexisting goitre, 10 had autoimmune thyroiditis, and 21 had hyperthyroidism [4]. A recent study suggested that the coexistence rate of hyperparathyroidism and thyrotoxicosis was much higher than previously reported [5]. Abboud et al. reported that the prevalence of concomitant hyperthyroidism was 13.5% in patients admitted for surgery for hyperparathyroidism [6]. In cases with concomitant hyperthyroidism and PHPT, the aetiology for hyperthyroidism is most commonly Graves’ disease [7–12]. An aetiology of TSH-secreting pituitary adenoma [13] or high-function adenoma [14] is rarely reported. In addition, concomitant PHPT with toxic nodular goitre is very rare. Only two cases have been reported in the literature [15, 16]. Here, we present a rare case of the coexistence of primary hyperparathyroidism with giant toxic nodular goitre that was missed for many years and successfully treated with ultrasound-guided parathyroid adenoma MWA.

The patient has been suffering from urinary stones for many years and had a low trauma fracture and biochemical abnormalities of hypercalcaemia and hypophosphatemia in 2013. These symptoms suggest the possibility of hyperparathyroidism. Unfortunately, as hyperthyroidism itself could lead to increased bone resorption [17], the hypercalcemia was ignored at that time. Therefore, he did not receive a PTH examination, and this diagnosis was missed at that time. Hyperthyroidism and hyperparathyroidism coexisted in this patient for nearly 10 years.

Accurate localization plays a crucial role in the treatment and prognosis of the coexistence of hyperparathyroidism and toxic nodular goitre. An MIBI scan has a sensitivity of 70-90% for detecting parathyroid adenoma, making it the most precise method for the location of a single parathyroid adenoma diagnosis [18]. The retention of this tracer in parathyroid lesions is presumably related to the presence of mitochondria-rich oxyphil cells. However, false-positives and false negatives exist in the MIBI scan results. It has been reported that false-positive MIBI scans can be attributed to nodular goitre, thyroid adenomas and metastatic thyroid cancer [19]. False-positive results occur when structures with mitochondria-rich tissues other than a parathyroid adenoma take up and concentrate the technetium 99mTc-labelled radiopharmaceutical to a higher degree than surrounding tissues. The presence of cysts in parathyroid proliferative lesions, small size and multiple numbers of parathyroid lesions and ectopic glands also contribute to false negatives in parathyroid MIBI scans [20]. In the former examination of this patient, the increased MIBI uptake due to the toxic nodular goitre accounts for the misdiagnosis, suggesting that false-positive MIBI results should be considered when hyperparathyroidism and nodular goitre coexist. In addition, the hot spot caused by the toxic nodular goitre in the MIBI scan may cause other weak focal to be undetectable, which leads to false-negative results.

Moreover, the misdiagnosis of this patient was previously related to the selection of MIBI scan technologies. SPECT/CT provides both functional and anatomical data and has proven to be superior to planar SPECT for the precise localization and characterization of areas of marked uptake of radiotracers by MIBI corresponding to abnormal parathyroid glands [21]. In this case, planar imaging may lead to focal overlap, resulting in false negatives before surgery. Therefore, when no lesion was observed by planar imaging, ultrasound examination or intraoperative exploration, we selected SPECT/CT at the final admission. Eventually, SPECT/CT found a suspicious lesion on the right side of the thyroid gland. Moreover, CEUS confirmed the presence of a blood-rich nodule at this location. This suggested a parathyroid adenoma and supported our location diagnosis.

It is currently believed that patients with recurrent and persistent hyperparathyroidism should have at least two preoperative localization methods to locate the diseased parathyroid in the same anatomical area before reoperation to reduce the risk of surgery [21]. Ultrasound and MIBI scans are still established techniques that are commonly utilized as first-line modalities [22]. Nevertheless, ultrasound may miss some small and deep parathyroid lesions and requires more empirical value. For this patient, to improve the diagnostic accuracy, we reexamined the patient based on SPECT/CT combined with ultrasound and CEUS.

The current treatment strategies for PHPT mainly include surgery and ultrasound-guided percutaneous thermal ablation. In recent years, thermal ablation, such as MWA and radiofrequency ablation (RFA), has been proven effective in inactivating parathyroid nodules and normalizing serum PTH and calcium levels [22]. A recent prospective study reported that MWA and surgical resection had similar cure rates in PHPT [23]. Reoperation for PHPT carries an increased risk for cure failure and incidence of complications, as postoperative scar formation and normal anatomical changes can increase the complexity and risks of parathyroid gland resection. This aggravates complications, such as recurrent laryngeal nerve injury and hypoparathyroidism [24, 25]. Therefore, ultrasound-guided MWA was selected. The serum PTH and calcium levels returned to their normal ranges after MWA, and CEUS further indicated the success of the treatment strategy.

In addition, the patient had a history of severe osteoporosis with bone calcium loss and vitamin D deficiency for several years. The occurrence of ‘hungry bone syndrome’ which causes hypocalcaemia should be considered after treatment, and the degree of preoperative osteoporosis and the serum calcium, phosphorus, PTH and vitamin D levels may be related to the incidence and duration of hypocalcaemia. In addition, the presence of vitamin D deficiency could also lead to secondary elevated PTH levels. It has been previously reported that preoperative supplementation with vitamin D under the monitoring of blood and urine calcium is beneficial to reducing PTH levels and reducing the incidence of severe hypocalcaemia in postoperative patients [26, 27]. However, to avoid aggravating hypercalcemia, this patient was not administered vitamin D promptly before surgery, which deserves further reflection. In the postoperation period, vitamin D supplementation was administered. Additionally, it is also for the sake of avoiding vitamin D deficiency induced normo-calcemic parathormone elevation (NPE) status after treatment [28, 29].

## Data Availability

Not applicable.

## Abbreviations

MWA: Microwave ablation
PTH: Parathyroid hormone
PHPT: Primary hyperparathyroidism
CEUS: Contrast-enhanced ultrasound
TSH: Thyroid stimulating hormone
TRAb: Thyrotropin receptor antibody
MRI: Magnetic resonance imaging
CT: Computed tomography
SPECT/CT: Single-photon emission computed tomography combined with computed tomography
RFA: Radiofrequency ablation
